# Noncoding RNA-mediated regulation of pyroptotic cell death in cancer

**DOI:** 10.3389/fonc.2022.1015587

**Published:** 2022-10-31

**Authors:** Man Wang, Yuan Zhang, Wenguang Chang, Lei Zhang, Konstantinos N. Syrigos, Peifeng Li

**Affiliations:** ^1^ Institute for Translational Medicine, The Affiliated Hospital of Qingdao University, College of Medicine, Qingdao University, Qingdao, China; ^2^ Third Department of Internal Medicine and Laboratory, National & Kapodistrian University of Athens, Athens, Greece

**Keywords:** pyroptosis, noncoding RNAs, miRNAs, lncRNAs, circRNAs, cancer pathogenesis

## Abstract

Pyroptosis is a newly discovered form of programmed cell death, which is manifested by DNA fragmentation, cell swelling, cell membrane rupture and leakage of cell contents. Previous studies have demonstrated that pyroptosis is tightly associated with the initiation and development of various cancers, whereas the molecular mechanisms underlying pyroptosis remain obscure. Noncoding RNAs (ncRNAs) are a type of heterogeneous transcripts that are broadly expressed in mammalian cells. Owing to their potency of regulating gene expression, ncRNAs play essential roles in physiological and pathological processes. NcRNAs are increasingly acknowledged as important regulators of the pyroptosis process. Importantly, the crosstalk between ncRNAs and pyroptosis affects various hallmarks of cancer, including cell growth, survival, metastasis and therapeutic resistance. The study of the involvement of pyroptosis-associated ncRNAs in cancer pathobiology has become a hot area in recent years, while there are limited reviews on this topic. Herein, we provide an overview of the complicated roles of ncRNAs, mainly including microRNAs (miRNAs), long noncoding RNAs (lncRNAs) and circular RNAs (circRNAs), in modulating pyroptosis, with a focus on the underlying mechanisms of the ncRNA-pyroptosis axis in cancer pathogenesis. Finally, we discuss the potential applications and challenges of exploiting pyroptosis-regulating ncRNAs as molecular biomarkers and therapeutic targets in cancer.

## 1 Introduction

Cancer remains a principal contributor to morbidity and mortality worldwide (1, 2). As a key hallmark of cancer, the ability to evade cell death not only promotes the genesis of cancer, but also leads to the acquisition of metastatic potential and therapeutic resistance (3). Cancer cells have evolved multiple strategies to circumvent or block cell death pathways (4). Therefore, inducing cancer cell death is undoubtedly an important mechanism responsible for tumor clearance. These are several known forms of cell death, including apoptosis, autophagy, necrosis and pyroptosis (5). Among these cell death types, pyroptosis is a newly discovered mode of programmed cell death and is characterized by pore formation in the cell membrane, cell swelling, cell lysis and the release of intracellular components (6). Pyroptosis is predominantly mediated by various inflammasomes that are capable of recognizing endogenous or exogenous danger signals and inducing the leakage of pro-inflammatory factors including interleukin (IL)-1β and IL-18 (7). It is widely accepted that pyroptosis functions as a critical defense mechanism that protects the host against microbial infections (8). Recent studies have shown that pyroptosis is tightly associated with the pathogenesis of various diseases, such as cancers, cardiovascular and inflammatory diseases (9–11). At present, pyroptosis is a new frontier in cancer research. Concerted efforts have been made to uncover the regulatory mechanisms of pyroptosis and the profound impact of pyroptosis on cancer pathogenesis.

For decades, protein-coding genes were thought to be crucial players in gene regulatory networks. With the development of high-throughput sequencing technology, it turns out that protein-coding genes only account for around 2% of the human genome, and over 90% of the genome is transcribed as noncoding RNAs (ncRNAs) that do not code for proteins (12). Based on their biogenetic modes and functions, ncRNAs are divided into diverse subgroups that encompass microRNAs (miRNAs), small interfering RNAs (siRNAs), long noncoding RNAs (lncRNAs) and circular RNAs (circRNAs) (13). The discovery of tens of thousands of ncRNAs has challenged the notion that non-protein-coding genes are nonfunctional and has revolutionized the field of RNA biology. Mounting evidence has confirmed that ncRNAs constitute a hidden layer of internal signals that control gene expression at multiple levels (14). Accordingly, ncRNAs are involved in various cellular processes under both physiological and pathological conditions (15, 16). A large number of studies have manifested that ncRNAs can act as tumor suppressors or oncogenes in cancer (17–19). It has been proven that ncRNAs affect various aspects of cancer progression by interfering with the pyroptosis pathway. Particularly, ncRNAs directly target key components of the pyroptosis pathway such as gasdermins (GSDMs) and inflammasomes (20, 21). NcRNAs also regulate upstream signaling proteins of the pyroptosis pathway (22, 23). It is worth noting that the mechanisms underlying the role of ncRNAs in pyroptosis regulation may be complex and multifaceted, and thus remain to be delineated. The association of ncRNA-mediated pyroptotic signaling networks with carcinogenesis has become one of the hottest topics in biomedical science, which is worthy of systematic and detailed exploration.

In this review, we summarize the recent findings pertaining to the contributions of various miRNAs, lncRNAs and circRNAs to pyroptosis regulation in the contexts of cancer initiation and development. We also highlight the underlying mechanisms through which these ncRNA subtypes modulate pyroptosis in different cancers. In addition, we discuss potential future research directions in the related field. An in-depth perception of the relationship between ncRNA-mediated pyroptosis and cancer will be beneficial for comprehensively elucidating the molecular mechanisms involved in carcinogenesis and provide important clues for developing novel therapeutic strategies for cancer treatment.

## 2 Noncoding RNAs

With the advent of next-generation sequencing technology, it has now been realized that a large proportion of the eukaryotic genome is transcribed into ncRNAs (24). NcRNAs are a type of RNA molecules that commonly lack protein-coding potential. Based on their length, ncRNAs can be roughly divided into two categories: small ncRNAs (sncRNAs) with a length shorter than 200 nucleotides (nt) and lncRNAs with a length longer than 200 nt (25). SncRNAs include miRNAs, siRNAs and piwi-interacting RNAs (piRNAs).

### 2.1 MicroRNAs

Among the common types of sncRNAs, miRNAs are the most extensively studied. miRNAs are small, highly conserved ncRNAs approximately 21-23 nt in length (26). miRNAs act as master modulators of gene expression and play a critical role in various biological, physiological and pathological processes, such as development, cell proliferation, differentiation, immune regulation and cancer progression (27). The biogenesis of miRNAs has been preliminarily characterized. The primary transcripts of miRNA genes (pri-miRNAs) are processed into precursor miRNAs (pre-miRNAs) by the microprocessor complex consisting of Drosha and DiGeorge Syndrome Critical Region 8 (DGCR8) in the nucleus (28). Following nuclear processing, pre-miRNAs are exported to the cytoplasm under the action of the nuclear transporter exportin-5. In the cytoplasm, pre-miRNAs are further cleaved by Dicer, contributing to the generation of miRNA duplexes. The miRNA duplex unwinds and the mature miRNA is successively loaded into an Argonaute protein of the RNA-induced silencing complex (RISC) (29). The mature miRNA functions as a guide by base-pairing with complementary seed sites within the 3’ untranslated region (UTR) of mRNAs, resulting in either mRNA degradation or translation repression. The extent of complementarity between miRNA and target mRNAs determines which silencing manner acts.

### 2.2 Long noncoding RNAs

LncRNAs are mostly transcribed by RNA polymerase II (Pol II) at several loci of the genome (30). The resulting lncRNAs undergo post-transcriptional processing, such as 5’-end capping, alternative splicing and polyadenylation, similarly to protein-coding mRNAs. According to their genomic origins, lncRNAs can be generally categorized into five classes: antisense, bidirectional, intronic, long intergenic and sense (31). Many lncRNAs are inefficiently processed and retained in the nucleus, while other lncRNAs that harbor at least one exon are transported to the cytoplasm by nuclear RNA export factor 1 (NXF1). The expression of lncRNAs can be modulated by both transcriptional and epigenetic factors. Canonical factors including pre-initiation complex, mediator complex, and transcription elongation complex are dispensable for transcription of many lncRNA genes (32). Several transcription factors, including Nanog, sex-determining region Y-box2 (Sox2), tumor protein 53 (TP53) and zinc finger protein 143 (ZNF143), participate in the transactivation of lncRNA gene expression (33). DNA methylation and histone modifications are epigenetic mechanisms contributing to lncRNA deregulation (34). Hyper-methylated lncRNA genes could be re-expressed by DNA methyltransferase (DNMT) inhibitors (e.g., 5-azadC) and histone deacetylase (HDAC) inhibitors (e.g., 4-phenylbutyric acid and trichostatin) (35, 36). Particularly, lncRNA genes have higher DNA methylation levels around transcription start site (TSS) than protein-coding genes regardless of their expression status, demonstrating the discrepancy in epigenetic regulatory mechanisms between lncRNA genes and protein-coding genes (37). Further studies are required to uncover the precise mechanisms underlying the regulation of lncRNA expression.

LncRNAs exhibit tissue- and cell type-specific expression patterns and are expressed at lower levels than protein-coding genes (38). LncRNAs have the ability to interact with DNAs, RNAs and proteins. Thus, lncRNAs operate as prominent regulators of gene expression by affecting the chromatin state, pre-mRNA splicing, mRNA stability, transcription, translation and post-translational regulation (39). In terms of molecular actions, lncRNAs can be grouped into four archetypes: decoy, guide, scaffold and signal (40). Decoy lncRNAs act as molecular sponges for various regulatory factors, such as chromatin modifiers, RNA-binding proteins (RBPs), RNA molecules and transcription factors, resulting in gene activation or silencing (41). Guide lncRNAs are able to control cellular signaling events and gene expression by directing transcriptional and epigenetic regulatory factors to particular genome locations (42). Scaffold lncRNAs serve as a central platform for the assembly of multi-component complexes, such as ribonucleoprotein (RNP) complexes that target certain genomic loci or gene promoters (43). Thus, scaffold lncRNAs play a role in regulation of chromatic dynamics and gene expression. Signal lncRNAs are expressed in a specific spatiotemporal mode and can be used as molecular signals to dominate transcription in response to multiple stimuli (44). The expression and existence of signal lncRNAs are an indicator of transcriptional activity and represent an active signaling event. Because of their versatile functions, lncRNAs have been widely involved in a variety of biological processes, including cell proliferation, differentiation and death (45). Intriguingly, lncRNAs can encode functional short peptides, which are engaged in muscle-related functions and cancer pathogenesis (46).

### 2.3 Circular RNAs

CircRNAs are a novel class of lncRNA species with covalently closed loop structures (47). CircRNAs were originally regarded as accidental byproducts resulting from transcriptional errors or splicing intermediates (48). With the advances in high-throughput sequencing and bioinformatics approaches, it is now realized that circRNAs are abundantly expressed, highly stable and widely evolutionary conserved across various species (49, 50). CircRNAs exhibit cell type-, tissue- and developmental stage-specific expression patterns, suggesting their important roles in multiple biological processes. Due to the lack of 5’ cap structures and 3’ polyadenosine (poly (A)) tails, circRNAs are resistant to mRNA-degrading enzymes and more stable than linear RNAs (51). Therefore, circRNAs are more suitable to be used as diagnostic biomarkers and therapeutic targets compared with other types of RNAs.

The formation of circRNAs is primarily dependent on a special form of alternative splicing termed back-splicing, in which the 3’ end of an exon connects to the 5’ end of its own or an upstream exon through a 3’, 5’-phosphodiester bond (52). Canonical splicing sites and spliceosome machinery are required for circRNA production (53). According to their composition, circRNAs can be divided into three categories: exonic circRNAs (ecircRNAs), exon-intron circRNAs (EIciRNAs) and circular intronic RNAs (ciRNAs). Among them, ecircRNAs, comprising one or more exons, occupy the vast majority of circRNAs and are localized in the cytoplasm (51). EIciRNAs and ciRNAs predominantly reside in the nucleus. Several circRNA biogenetic models, including lariat-driven circularization, intron pairing-driven circularization and RBP-dependent circularization, have been proposed (54). Lariat-driven circularization is referred to as the exon-skipping mechanism. The exon-skipping event during pre-mRNA splicing leads to the production of a lariat structure containing exons and introns (55). The removal of introns is accomplished by internal cleavage of the lariat precursor, through which circRNAs are formed. Likewise, ciRNAs are created from intronic lariat precursors, and their biosynthesis principally relies on a consensus RNA motif containing a 7 nt GU-rich element close to the 5’ splice site and an 11 nt C-rich element near the branchpoint site to escape the process of intron debranching and exonucleolytic degradation (56). Intron pairing-driven circularization is also known as direct back-splicing pathway. The base pairing of inverse-repeating or complementary sequences within introns on both sides of the exon results in the generation of a circular structure (57). RBPs play an important role in circRNA biogenesis (58). During RBP-dependent circularization, RBPs act as a bridge linking the upstream and downstream introns close together by binding to specific intronic motifs, which facilitates the formation of circRNAs. In these models, ecircRNAs or EIciRNAs are synthesized according to whether or not internal introns are completely removed.

The function mechanisms of circRNAs have been gradually appreciated in recent years. The most outstanding function of circRNAs is their role as miRNA sponges to modulate target gene expression. CDR1as is a representative example, which contains conserved miR-7 complementary sequences across many species (59). CDR1as was implicated in mammalian brain development and the pathobiology of cancer by sequestering miR-7 (60–62). Moreover, one circRNA can interact with various miRNAs, such as circHIPK3, which served as a molecular sponge for a list of miRNAs, including miR-29a, miR-29b, miR-124, miR-152, miR-193a, miR-338, miR-379, miR-584 and miR-654 (63). CircRNAs can act as protein decoys to affect cellular functions. For instance, circ-TNPO3 competitively interacted with insulin-like growth factor 2 mRNA-binding protein 3 (IGF2BF3) to attenuate its effect on the activation of the Myc/Snail axis (64). Thus, circ-TNPO3 played an inhibitory role in the proliferation and metastasis of gastric cancer (GC). CircRNAs can alter the expression of their parental genes. A specific example of this regulation is the EIciRNA. EIciRNAs associated with U1 small nuclear ribonucleoprotein (snRNP) *via* specific RNA-RNA interaction (65). The EIciRNA-U1 snRNP complex successively coupled with RNA Pol II at the promoter of parental genes to enhance their transcription. EIciRNAs served as dynamic protein scaffolds enabling the assembly of the transcribing RNA Pol II-U1 snRNP complex. Interestingly, some circRNAs harbor an internal ribosome entry site (IRES) or N^6^-methyladenosine modification, and thus they have the ability to encode functional peptides (66, 67). CircFNDC3B coded for a novel protein circFNDC3B-218aa, which exerted an inhibitory effect on the progression of colon cancer (68). Further exploration of circRNA-derived proteins is expected to broaden our understanding of biological functions of circRNAs.

## 3 Pyroptosis in cancer

Pyroptosis, also referred to as cellular inflammatory necrosis, is a newly discovered mode of programmed cell death mediated by GSDM family proteins. Membrane perforation, cell swelling, cell membrane rupture and the release of cell contents are critical characteristics of pyroptosis (69). An expanding number of studies have revealed that pyroptosis is tightly associated with the occurrence and development of cancer.

### 3.1 Core mechanisms of pyroptosis

**Figure 1:**
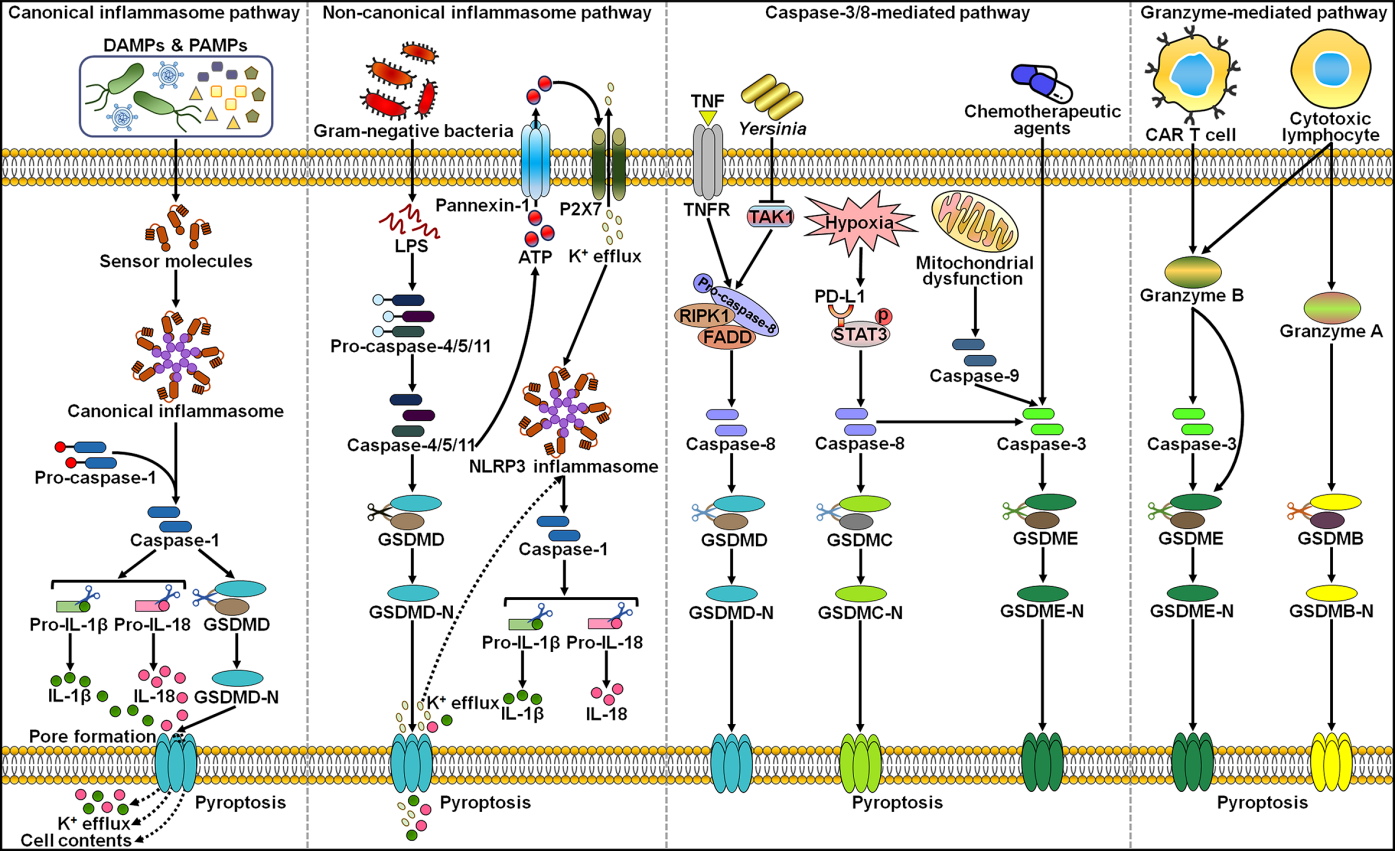
The main molecular mechanisms of pyroptosis. In the canonical inflammasome pathway, cellular signals (e.g. DAMPs and PAMPs) can activate the canonical inflammasome and capase-1. Active caspase-1 cleaves GSDMD to unleash its N-terminal domain (GSDMD-N), which perforates the cell membrane by forming oligomeric pores. These events lead to the loss of membrane integrity and cell lysis. In addition, caspase-1 cleaves pro-IL-1β and pro-IL-18 into their mature forms, which are released into the extarcellular milieu through the GSDMD-N pore. In the non-canoncial inflammasome pathway, cytosolic LPS from invading Gram-negative bacteria directly activates inflammatory caspase-4/5/11, thus evoking GSDMD-mediated pyroptosis. The GSDMD-N pore causes the efflux of potassium (K^+^), eventually inducing the assembly of NLRP3 inflammasome and caspase-1-dependent IL-1β and IL-18 secretion. In addition, acitve caspase-11 induces the opening of the pannexin-1 channel, and thus tiggers the extracellular release of ATP, which activates NLRP3 inflammasome *via* the P2X7 receptor. In caspase-8-mediated pathway, the pathogenic *Yersinia* infection results in inhibition of the TAK1 activity, motivating the RIPK1/caspase-8 pathway. Active caspase-8 cleaves GSDMD to produce GSDMD-N, resulting in pyroptotic cell death. Under hypoxia conditions, p-STAT3 physically binds to PD-L1 and favors its nuclear translocation, where PD-L1 enhances *GSDMC* gene expression. TNF-α-activated caspase-8 specifically shears GSDMC, liberating its N-terminal domain (GSDMC-N) that punches holes in the cell membrane and triggers pyroptosis. In capase-3-mediated pathway, chemotherapeutic agent-activated caspase-3 converts GSDME into GSDME-N, which perforates the cell membrane to initiate pyroptosis. In the granzyme-mediated pathway, CAR T cells prompt caspase-3/GSDME-dependent pyroptosis in target cells by secreting granzyme B. Futhermore, granzyme B directly cuts GSDME to drive pyroptosis. In addition, cytotoxic lymphocyte-derived granzyme A can activate the pyroptosis signaling pathway *via* specific cleavage of GSDMB. DAMPs, damage-associated molecular patterns; PAMPs, pathogen-associated molecular patterns; IL-1β, interleukin-1β; IL-18, interleukin-18; GSDMD, gasdermin D; GSDMD-N, the N-terminal domain of gasdermin D; LPS, lipopolysaccharide; ATP, adenosine triphosphate; P2X7, purinergic receptor P2X ligand-gated ion channel 7; TNF, tumor necrosis factor; TNFR, tumor necrosis factor receptor; RIPK1, receptor-interacting protein kinase 1; FADD, Fas-associated protein with death domain; TAK1, transforming growth factor-β (TGF-β)-activated kinase 1; PD-L1, programmed death-ligand 1; STAT3, signal transducer and activator of transcription 3; GSDMC, gasdermin C; GSDMC-N, the N-terminal domain of gasdermin C; GSDME, gasdermin E; GSDME-N, the N-terminal domain of gasdermin E; CAR, chimeric antigen receptor; GSDMB, gasdermin B; GSDMB-N, the N-terminal domain of gasdermin B.

So far, several different pathways were identified to activate the action of pyroptosis (**Figure 1**). Generally, pyroptosis is induced in an inflammasome-dependent or independent manner (70). The inflammasome-dependent mechanisms include caspase-1-mediated canonical and caspase-4/5/11-mediated non-canonical pathways, while the inflammasome-independent mechanisms include caspase-3-, caspase-8- and granzyme-mediated pathways.

The canonical inflammasome is a cytoplasmic polyprotein complex composed of a sensor protein, an adaptor protein termed apoptosis-associated speck-like protein containing a caspase activation and recruitment domain (ASC) and an effector protein pro-caspase-1 (71, 72). Sensor proteins nucleotide-binding oligomerization domain (NOD)-like receptor (NLR) family pyrin domain-containing 1 (NLRP1), NLRP3, NLR family caspase activation and recruitment domain (CARD)-containing 4 (NLRC4), absent in melanoma 2 (AIM2) and pyrin can form canonical inflammasomes. The GSDM family consists of GSDMA, GSDMB, GSDMC, GSDMD, GSDMDE (DFNA5) and DFNB59 (Pejvakin, PJVK) in humans (73). It is worth mentioning that mice possess three GSDMA subtypes (GSDMA1-3) and four GSDMC subtypes (GSDMC1-4) but do not have GSDMB. Except for DFNB59, all members of the GSDM family are composed of N-terminal and C-terminal domains, and the N-terminal domain acts as the pyroptosis executor. In caspase-1-mediated canonical inflammasome pathway, the assembly of inflammasomes is triggered upon the recognition of diverse stimuli, such as damage-associated molecular patterns (DAMPs), pathogen-associated molecular patterns (PAMPs) and microbial infections, by distinct sensor proteins. Afterwards, sensor proteins homo-oligomerize and recruit pro-caspase-1 through homotypic interactions or through the adaptor protein ASC. Combination of pro-caspase-1 with the inflammasome causes the initiation of its autocatalytic activity, culminating in the production of catalytically active caspase-1 through self-cleavage (74). Active caspase-1 cleaves GSDMD at its middle linker to form an N-terminal pore-forming domain (GSDMD-N) and a C-terminal repressor domain. GSDMD-N then oligomerizes and perforates the cell membrane, leading to water influx, cell swelling and osmotic lysis. Caspase-1 processes the precursors of pro-inflammatory cytokines IL-1β and IL-18 into their mature forms. Bioactive IL-1β and IL-18 are liberated through the GSDMD-N pore. Pyroptosis can cause the leakage of DAMPs including high-mobility group box 1 (HMGB1) and lactate dehydrogenase (LDH), further augmenting inflammation and recruiting immune cells to the tissue (75).

In the non-canonical inflammasome pathway, intracellular lipopolysaccharide (LPS) directly binds the N-terminal CARD of human caspase-4/5, or mouse homologue casaspe-11 (76, 77). Activated caspase-4/5/11 subsequently cleave the executor protein GSDMD to unleash its N-terminal domain (GSDMD-N), which transfers to the cell membrane and induces lytic cell death by forming oligomeric pores. Caspase-4/5/11 do not directly induce maturation of IL-1β and IL-18. The GSDMD-N pores result in potassium (K^+^) efflux that activates the NLRP3/caspase-1 pathway, contributing to the hydrolysis and release of IL-1β and IL-18 (78, 79). Following cytosolic LPS stimulation, active caspase-11 specifically cleaved the pannexin-1 channel followed up by release of cellular adenosine triphosphate (ATP) (80). The increased extracellular ATP activated the purinergic receptor P2X ligand-gated ion channel 7 (P2X7), which allowed K^+^ efflux and thus led to the assembly of NLRP3 inflammasome.

Once activation by chemotherapy drugs, caspase-3 mediates the cleavage of GSDME in GSDME-expressing cancer cells to liberate its N-terminal domain (GSDME-N), hence inducing cancer cell pyroptosis (81, 82). In mouse macrophages, pathogenic *Yersinia* suppressed the activity of transforming growth factor-β (TGF-β)-activated kinase 1 (TAK1) *via* the effector protein YopJ, evoking the receptor-interacting protein kinase 1 (RIPK1)/caspase-8 pathway (83). Activated caspase-8 cleaved GSDMD into GSDMD-N, which punched holes in the cell membrane to induce pyroptosis. Programmed death-ligand 1 (PD-L1) switched tumor necrosis factor-α (TNF-α)-induced apoptosis to pyroptosis in breast cancer (BC) cells (70). Mechanistically, hypoxic stress induced phosphorylated signal transducer and activator of transcription 3 (p-STAT3)-mediated nuclear translocation of PD-L1, enhancing *GSDMC* gene transcription. TNF-α-activated caspase-8 specifically sheared GSDMC to generate GSDMC-N, which drilled holes in the cell membrane to drive pyroptotic cell death. Furthermore, antibiotic chemotherapeutic drugs could prompt caspase-8/GSDMC-induced pyroptosis in BC cells.

Emerging evidence has refined the notion that pyroptosis could only be mobilized by caspases. The granzyme-mediated pyroptosis pathway has been reported in several studies. Chimeric antigen receptor (CAR) T cells were found to expeditiously activate caspase-3 in target cells by secreting granzyme B (GzmB) (84). Active caspase-3 cleaved GSDME to unleash its pore-forming N-terminal domain, which led to excessive pyroptosis. Another study showed that GzmB directly cut GSMDE at the same site as caspase-3 and promoted pyroptosis in GSDME-expressing cancer cells, which further aggrandized the cytotoxic actions of antitumor killer cells (85). Zhou et al. (86) revealed that cytotoxic lymphocyte-derived GzmA was able to hydrolyze GSDMB at the Lys299/Lys244 site within the interdomain linker, unmasking the pore-forming activity of GSDMB and finally causing extensive pyroptosis. In addition, such immune effector mechanism potentiated cytotoxic T lymphocyte (CTL)-mediated tumor regression *in vivo*.

Pyroptosis constitutes a pivotal component of host innate immune system and performs essential functions in microbial clearance and anticancer effects. Although pyroptosis has been increasingly studied in recent years, there are many gaps in our current knowledge. The regulatory mechanisms of pyroptosis are still in need of detailed study. Caspase-1 and caspase-11 shear GSDMD through the identical chemical mechanism, but only caspase-1 can activate IL-1β and IL-18. It is confusing why capase-11 cannot induce the maturation of IL-1β and IL-18. GSDMA-E have been substantiated to function as the executors of pyroptosis. These GSDM family members are implicated in the pyroptosis process under distinct circumstances. It is equivocal whether GSDM family proteins play overlapping roles in inflammation and carcinogenesis. Much work is necessary to illuminate certain aspects of the non-canonical inflammasome mechanism. LPS is considered as an important substrate that motivates the non-canonical inflammasome pathway. It is puzzling whether there are other substrates for the non-canonical inflammasome. There is an urgent need to characterize the structural and mechanistic basis of the interplay between the non-canonical inflammasome and GSDMs. More efforts are required to determine critical factors involved in the regulation of pyroptosis. The endosomal sorting complex required for transport-III (ESCRT-III) system is responsible for the repairment of GSDM pores (87). The role of ESCRT-III-mediated membrane repair pathway in GSDM-executed pyroptosis needs further investigation. It is essential to explore the fate of GSDM pores during the pyroptosis process. The interconnection between pyroptosis and other death pathways (e.g., apoptosis and necroptosis) may represent an interesting research direction. Above all, the functions and mechanisms of pyroptosis warrant more in-depth studies. In addition, further studies to seek activators of the pyroptosis pathway may open up new therapeutic avenues for many types of diseases including immune-related diseases and cancers.

### 3.2 The regulatory roles of pyroptosis in cancer pathobiology

The relationship between the pyroptosis pathway and cancer pathogenesis is complicated. The role of pyroptosis in cancer can vary depending on cancer cell type, genetic background, the duration and extent of pyroptosis. On one hand, pyroptosis suppresses the initiation and development of cancer. Overexpressed p53 inhibited the growth of non-small cell lung cancer (NSCLC) by inducing NLRP3 inflammasome-dependent pyroptosis (88). The absence of active inflammasomes promoted azoxymethane/dextran sulfate sodium (AOM/DSS)-induced colorectal carcinogenesis in mice, suggesting that inflammasomes acted as a negative modulator of cancer formation (89–91). GSDME-executed pyroptosis enhanced the anticancer efficacy of the chemotherapeutic drug carboplatin in retinoblastoma cells (92). Remarkably, pyroptosis exerts an anticarcinogenic role by activating immune responses. GSDME-mediated pyroptosis enhanced the cytotoxic effects of cisplatin on NSCLC cells by releasing chemokines to attract tumor-infiltrating T cells (93). HMGB1 released from pyroptotic melanoma cells induced the activation of dendritic cells (DCs) and antitumor T cells, which in turn prevented melanoma growth (94). On the other hand, pyroptosis facilitates the onset and progression of cancer in various ways. The main components of the pyroptosis pathway have a role in promoting cancer pathogenesis. For instance, upregulation of the pyroptosis effector GSDMC induced by TGF-β receptor type II (TGFBRII) favored colorectal carcinogenesis *in vivo* (95). Pyroptosis may contribute to the establishment of a favorable niche for cancer progression. The activation of pyroptosis caused the liberation of various inflammatory mediators (e.g., IL-1 and IL-18), which fueled cancer development through diverse mechanisms including upregulation of proangiogenic factors and adhesion molecules, regulation of inflammatory tumor microenvironment and disruption of antitumor immunity (96). Moreover, pyroptosis can regulate cancer progression by releasing tumor-promoting molecules. Reportedly, GSDME-mediated pyroptosis supported the development of colorectal cancer (CRC) *via* the discharge of HMGB1, which promoted proliferating cell nuclear antigen (PCNA) expression and cancer cell proliferation (97).

The mechanisms underlying the dual roles of pyroptosis in cancer have yet to be thoroughly elucidated. The key components of the pyroptosis pathway, inflammasomes, GSDMs and pro-inflammatory cytokines, are closely associated with cancer biology (98). Notably, a multitude of components play overlapping roles in diverse pathways mediating pyroptosis. Hence, addressing the overall effect of each pathway on cancer development, rather than the individual effect of single component, may be helpful to completely understand the regulatory role of pyroptosis in cancer. Activating pyroptosis in cancer cells is proposed to represent a novel alternative for conventional cancer treatments. Continual study of the pyroptosis mechanism in different types of cancers, as well as of the upstream and downstream factors of the pyroptosis pathway, would provide new opportunities to improve existing cancer treatments. Pyroptosis causes the release of numerous cellular contents to elicit robust inflammatory responses and extensive infiltration of immune cells in the tumor microenvironment. Conversely, enhanced lymphocyte infiltration prompts cancer cell pyroptosis, constituting a positive feedback to augment anticancer effects (99). The reciprocal interaction between the pyroptosis pathway and antitumor immunity deserves more attention. Pyroptosis-based therapeutics could be used in combination with immunotherapy to achieve systemic control of cancer. However, conventional anticancer therapies have the potential to trigger pyroptotic cell death in immune cells, leading to damage of antitumor immunity. Therefore, it is important to diminish adverse effects of current treatments by preventing immune cell pyroptosis. There is a need for new approaches that specifically induce pyroptosis in cancer cells without impairing antitumor immune cells. *GSDME* is an epigenetically silenced tumor suppressor gene in most cancer cells (81). It is suggestive that specific induction of pyroptosis in cancer cells by activating GSDME may provide an effective approach to enhance antitumor immunity. Additionally, intensive experimental and clinical studies are required to evaluate the potential application of pyroptosis-based therapeutics.

## 4 Noncoding RNA-mediated pyroptosis in cancer

**Figure 2:**
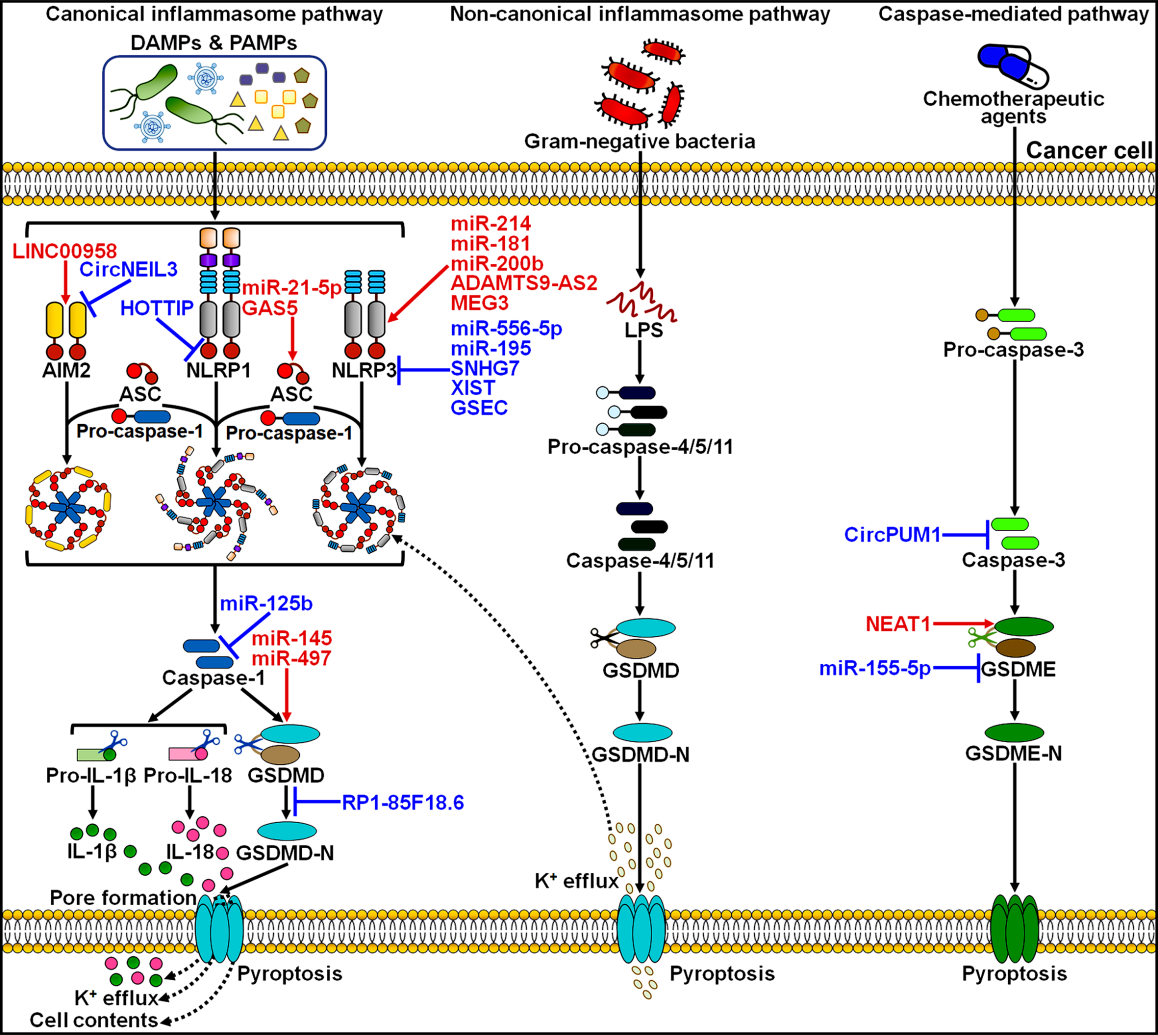
Overview of ncRNAs involved in the regulation of the pyroptosis pathways in cancer. LINC00958 was found to initiate AIM2 inflammasome-dependent pyroptosis. By contrast, circNEIL3 prevented DNA damage-induced AIM2 inflammasome activaiton, thereby restraining the pyroptosis process. HOTTIP was capable of suppressing NLRP1 inflammasome-mediated pyroptosis. miR-21-5p and GAS5 could activate the canonical inflammasome pathway by regulating ASC. miR-214, miR-181, miR-200b, ADAMTS9-AS2 and MEG3 were reported to facilitate the assembly of NLRP3 inflammasome, whereas miR-556-5p, miR-195, SNHG7, XIST and GSEC exerted the opposite action. miR-125b limited the activity of caspase-1, hence blocking the canonical inflammasome pathway. miR-145 and miR-497 drove the pyroptosis pathway by promoting GSDMD expression or activation. However, RP1-85F18.6 inhibited GSDMD cleavage and thus repressed pyroptoic cell death. CircPUM1 negatively regulated caspase-3 to interrupt the related pyroptosis pathway. NEAT1 and miR-155-5p had opposite effects on GSDME-executed pyroptosis. DAMPs, damage-associated molecular patterns; PAMPs, pathogen-associated molecular patterns; AIM2, absent in melanoma 2; ASC, apoptosis-associated speck-like protein containing a caspase activation and recruitment domain; NLRP1, nucleotide-binding oligomerization domain (NOD)-like receptor (NLR) family pyrin domain-containing 1; NLRP3, nucleotide-binding oligomerization domain (NOD)-like receptor (NLR) family pyrin domain-containing 3; GSDMD, gasdermin D; GSDMD-N, the N-terminal domain of gasdermin D; IL-1β, interleukin-1β; IL-18, interleukin-18; LPS, lipopolysaccharide; GSDME, gasdermin E; GSDME-N, the N-terminal domain of gasdermin E.

Various types of ncRNAs, including miRNAs, lncRNAs and circRNAs, can regulate pyroptotic cell death in cancer, which opens a new window to better understand the pyroptosis mechanism (**Figure 2**). The pyroptosis pathway is involved in the onset and progression of cancer. The interaction between ncRNAs and pyroptosis has recently become a new focus of research within the field of oncology.

### 4.1 Regulation of pyroptosis by miRNAs in cancer

Many studies have addressed the evidence of miRNA-mediated regulation of pyroptosis. miRNAs can either promote or suppress cancer cell pyroptosis *via* regulating different genes and signaling pathways (**Table 1**).

**Table 1:** NcRNA-mediated pyroptosis in cancer pathogenesis.

NcRNA	Cancer type	Expression	Target	Effect on pyroptosis	Function	Reference
miR-214	Cervical cancer	↓ downregulated	NLRP3	Promotion	Retardation of cell proliferation	(20)
miR-145	Cervical cancer	↓ downregulated	GSDMD	Promotion	Retardation of cell proliferation	(100)
miR-181	Neuroblastoma	↓ downregulated	The SIRT1/PGC-1α/Nrf2 signaling	Promotion	Retardation of cell proliferation	(22)
miR-200b	Breast cancer	↓ downregulated	JAZF1	Promotion	Repression of cell growth	(101)
miR-497	Esophageal squamous cell carcinoma	↓ downregulated	PELP1	Promotion	Retardation of cell proliferation	(102)
miR-21-5p	Colorectal cancer	↑ upregulated	TGFBI	Promotion	Reduction of cell viability	(103)
miR-556-5p	Non-small cell lung cancer	↑ upregulated	NLRP3	Suppression	Attenuation of cell chemosensitivity	(104)
miR-195	Neuroblastoma	↑ upregulated	NLRX1	Suppression	Blockade of EV-A71-induced cell death	(105)
miR-155-5p	Triple-negative breast cancer	↑ upregulated	GSDME	Suppression	Attenuation of cell chemosensitivity	(106)
miR-125b	Nasopharyngeal carcinoma	↑ upregulated	FOXP3	Suppression	Acceleration of cell proliferation	(107)
LINC00958	Oral squamous cell carcinoma	↑ upregulated	miR-4306/AIM2	Promotion	Acceleration of cell proliferation	(108)
SNHG7	Hepatocellular carcinoma	↑ upregulated	miR-34a/SIRT1	Suppression	Acceleration of cell proliferation	(109)
ADAMTS9-AS2	Gastric cancer	↓ downregulated	miR-223-3p/NLRP3	Promotion	Enhancement of cell chemosensitivity	(110)
NEAT1	Colorectal cancer	↓ downregulated	miR-448/GSDME	Promotion	Enhancement of cell radiosensitivity	(21)
XIST	Non-small cell lung cancer	↑ upregulated	miR-335/SOD2	Suppression	Acceleration of cell proliferation	(111)
HOTTIP	Ovarian cancer	↑ upregulated	miR-148a-3p/Akt2	Suppression	Acceleration of cell proliferation	(112)
MEG3	Triple-negative breast cancer	↓ downregulated	NLRP3	Promotion	Enhancement of cell chemosensitivity	(113)
XIST	Non-small cell lung cancer	↑ upregulated	SMAD2	Suppression	Attenuation of cell chemosensitivity	(114)
GAS5	Ovarian cancer	↓ downregulated	Unknown	Promotion	Reduction of cell viability	(115)
RP1-85F18.6	Colorectal cancer	↑ upregulated	ΔNp63	Suppression	Promotion of cell proliferation and invasion	(116)
GSEC	Lung adenocarcinoma	↑ upregulated	NLRP3	Suppression	Acceleration of cell growth	(117)
CircNEIL3	Lung adenocarcinoma	↑ upregulated	miR-1184/PIF1	Suppression	Attenuation of cell radiosensitivity	(23)
CircPUM1	Esophageal squamous cell carcinoma	↑ upregulated	The AMPK signaling pathway	Suppression	Acceleration of cell growth	(118)

#### 4.1.1 Promotion of cancer cell pyroptosis by miRNAs

##### 4.1.1.1 Influence on the pyroptosis signaling pathways

miR-214 and NLRP3 were lowly expressed in cervical cancer patients and cervical cancer cells compared to their normal counterparts (20). Upregulation of miR-214 triggered caspase-1-mediated pyroptosis and suppressed the proliferation of cervical cancer cells by upregulating NLRP3. The underlying mechanisms by which miR-214 affected the expression of NLRP3 deserve further study. It remains to determine whether miR-214 negatively regulates the specific gene capable of inhibiting NLRP3 expression. Tanshinone IIA exerted inhibitory effects on cervical cancer progression (100). The expression level of miR-145 was elevated in tanshinone IIA-treated cervical cancer cells. The miR-145 inhibitor overtly restrained tanshinone IIA-induced pyroptosis, as evidenced by decreased levels of GSDMD, IL-β and IL-18. These findings provided preliminary evidence of a linkage between miR-145 and pyroptosis in cervical cancer. Silencing of miR-145 caused the downregulation of GSDMD, but the regulatory mechanism was elusive. miR-145 might act as a novel therapeutic target of the treatment of cervical cancer. However, the direct targets of both miR-214 and miR-145 remain undetermined. Accordingly, more studies are necessary to seek an explanation for miRNA-mediated pyroptosis in cervical cancer.

miRNAs exert regulatory roles in cancer cell pyroptosis by orchestrating key components of the pyroptosis pathway. miRNAs may bind to pyroptosis-related genes, and thus affect their expression. Credible evidence is still needed to validate this hypothesis. It is worth noting that miRNAs could indirectly affect the abundance of pyroptosis-related proteins by targeting some other genes. Therefore, further exploration is required to reveal how miRNAs regulate the pyroptosis signaling pathway. A more sophisticated understanding of the roles of miRNAs in pyroptosis regulation may be conducive to clarifying the complex molecular mechanisms that provide stringent regulation over the activation of pyroptosis.

##### 4.1.1.2 Modulation of intracellular signaling cascades

miR-181 showed upregulation in chlorpyrifos (CPF)-treated neuroblastoma cells (22). miR-181 significantly inactivated the sirtuin 1 (SIRT1)/peroxisome proliferator-activated receptor γ coactivator-1α (PGC-1α)/nuclear factor erythroid 2-related factor 2 (Nrf2) signaling cascade in CPF-treated neuroblastoma cells. Nrf2 inhibition brought about an increase in the levels of pyroptosis-related proteins including NLRP3, caspase-1, IL-1β and IL-18. Therefore, miR-181 promoted CPF-induced neuroblastoma cell pyroptosis through downregulation of the SIRT1/PGC-1α/Nrf2 signaling pathway. However, the detailed mechanism by which miR-181 regulated the activity of the SIRT1/PGC-1α/Nrf2 signaling warrants thorough exploration. Nrf2 may play multifaceted roles in NLRP3 inflammasome activation. Nrf2 was required for NLRP3 activation *via* regulation of ASC speck formation (119). On the other hand, Nrf2 activation has anti-inflammatory roles by upregulating antioxidative and reactive oxygen species (ROS)-detoxifying proteins (120). Since ROS is dispensable for NLRP3 inflammasome activation, Nrf2 dampens NLRP3 inflammasome activation through attenuation of intracellular ROS levels and oxidative stress. It is possible that miR-181-mediated Nrf2 inhibition contributes to ROS-induced NLRP3 inflammasome activation, but this assumption remains to be further validated. In addition, the crosstalk between Nrf2 and NLRP3 inflammasome is intricate. The reasons why Nrf2 regulates NLRP3 inflammasome activation are not completely understood. Continual investigations on the interconnection between Nrf2 and NLRP3 inflammasome will deepen our understanding of the regulatory mechanism associated with cancer cell pyroptosis.

Nobiletin, a polymethoxylated flavone present in citrus fruits, enhanced miR-200b expression in BC cells (101). miR-200b overexpression promoted pyroptosis of BC cells by increasing the expression of NLRP3, ASC, GSDMD, caspase-1, IL-1β and IL-18. Further study indicated that miR-200b negatively regulated the expression of the tumor promoter JAZF zinc finger 1 (JAZF1). Upregulation of JAZF1 partially diminished the pro-pyroptotic effect of miR-200b. JAZF1 serves as a repressor of TAK1 that is a pivotal kinase upstream of the nuclear factor-κB (NF-κB) signaling cascade (121). Correspondingly, miR-200b overexpression and JAZF1 silencing caused the activation of the NF-κB signaling pathway (101). The NF-κB inhibitor partially reversed the promoting effect of JAZF1 deficiency on pyroptotic cell death in BC. Blockade of the NF-κB signaling restrained the expression of pyroptosis-related proteins NLRP3, caspase-1 and GSDMD, and limited the production of pro-inflammatory cytokines IL-1β and IL-18 (122). In other words, NF-κB was a critical participant in orchestrating pyroptosis. Accordingly, miR-200b induced pyroptosis of BC cells through regulation of the JAZF1/NF-κB axis.

Proline-, glutamic acid- and leucine-rich protein 1 (PELP1) was an oncogene, and its upregulation was linked with the progression of esophageal squamous cell carcinoma (ESCC) (102). Metformin could trigger ESCC cell pyroptosis. Mechanistic investigation showed that metformin diminished PELP1 expression by upregulating miR-497. The miR-497 inhibitor caused the upregulation of PELP1, and overexpressed PELP1 suppressed metformin-induced GSDMD translocation from cytoplasm to cell membrane and GSDMD cleavage. *In vivo* experiment also showed that the miR-497/PELP1 axis served as a key mediator in ESCC cell pyroptosis induced by metformin. PELP1 can interact with histone and coactivator-associated arginine methyltransferase 1 (CARM1) by acting as a scaffolding protein (123, 124). The miR-497/PELP1 axis may control pyroptotic cell death in ESCC *via* the epigenetic mechanism, and future studies are needed to corroborate this speculation.

miR-21-5p exhibited a high expression trend in CRC tissues and cells compared to their normal counterparts (103). Overexpression of miR-21-5p enhanced pyroptosis and reduced viability of CRC cells by increasing the expression of caspase-1, active IL-1β and GSDMD-N and promoting the formation of ASC specks. TGF-β-induced protein (TGFBI) was a downstream target of miR-21-5p. Depletion of TGFBI caused the upregulation of caspase-1, GSDMD-N and IL-1β and the enhancement of ASC oligomerization. TGFBI acted as a tumor promoter in CRC. Notably, the mechanisms of action of TGFBI in pyroptosis is still elusive and thus need further study. This study presented evidence for the assembly and activation of NLRP3 inflammasome; however, the precise inflammasome sensor related to miR-21-5p-mediated cell pyroptosis warrants further verification.

Pyroptosis has been associated with multiple signal transduction cascades, including the Nrf2 and NF-κB pathways. miRNAs function as pivotal participants in cancer cell pyroptosis by controlling these signaling cascades. As far as is known, one miRNA is able to simultaneously regulate the expression of many genes. miRNAs may interfere with different signaling pathways that have synergistic or antagonistic effects on pyroptosis. The interplay between these pathways regulated by the same miRNA is poorly understood and necessitates further exploration. There is accumulating evidence on an important function performed by miRNAs in controlling numerous intracellular signaling pathways. miRNA-associated regulatory networks underlying pyroptosis may be exquisitely fine-tuned by highly orchestrated mechanisms in cancer. It is intriguing why the pro-pyroptotic or anti-pyroptotic signaling cascades that become activated by miRNAs take a central position in the regulation of cancer cell pyroptosis under certain circumstances.

#### 4.1.2 Inhibition of cancer cell pyroptosis by miRNAs

##### 4.1.2.1 Blockade of NLRP3 inflammasome activation

miR-556-5p was dramatically upregulated in cisplatin-resistant NSCLC (CR-NSCLC) tissues and cells compared to cisplatin-sensitive NSCLC (CS-NSCLC) tissues and cells (104). miR-556-5p negatively regulated NLRP3 expression (125). Overexpressed miR-556-5p enhanced cisplatin resistance in CS-NSCLC cells. Conversely, miR-556-5p ablation decreased cell viability and prompted pyroptotic cell death in cisplatin-treated CR-NSCLC cells by increasing NLRP3 expression. Consistently, miR-556-5p knockdown boost IL-1β and IL-18 secretion. These phenomena were alleviated by the pyroptosis inhibitor necrosulfonamide (NSA) or NLRP3 knockdown. In addition, miR-556-5p silencing also promoted apoptosis of cisplatin-treated CR-NSCLC cells, while impairment of pyroptotic cell death could reverse this effect. Thus, miR-556-5p ablation augmented cisplatin-induced CR-NSCLC cell apoptosis by enhancing NLRP3-dependent pyroptosis. These results suggested the existence of the reciprocal crosstalk between cell pyroptosis and apoptosis. Altogether, miR-556-5p inhibition increased susceptibility of NSCLC cells to chemotherapy through induction of pyroptosis, which might provide alternative therapeutic options to counteract chemotherapeutic resistance in NSCLC patients.

Enterovirus A71 (EV-A71) infection was previously verified to induce caspase-1-mediated pyroptosis in human neuroblastoma cells (126). Zhu et al. (105) further revealed the role of miRNAs in EV-A71 infection-triggered pyroptosis. They found that miR-195 was markedly downregulated in EV-A71-infected human neuroblastoma cells. NLR family member X1 (NLRX1), a member of the NLR family that localizes to mitochondria, acted as a negative modulator of antiviral signaling (127). NLRX1 might play an important in regulation of pathogen-induced pyroptosis by controlling NLRP3 inflammasome activation (128). miR-195 was proven to directly target NLRX1 (105). Knockdown of NLRX1 or ectopic expression of miR-195 inhibited EV-A71-caused pyroptosis in human neuroblastoma cells. It could be concluded that miR-195 modulated EV-A71-associated pyroptosis by targeting NLRX1. However, the accurate mechanism of NLRX1 in EV-A71-induced pyroptosis remains to be revealed. Moreover, the effect of NLRX1 on NLRP3 inflammasome activation should be defined in future studies.

miRNAs can directly or indirectly impact the activation of NLRP3 inflammasome. Since other inflammasomes (e.g., AIM2 and NLRP1) also take part in cell pyroptosis, the effect of miRNAs on inflammasome activation merits further intensive investigation. Due to the interaction between the apoptotic and pyroptotic pathways, it is expected that apoptosis-related miRNAs may have a role in curbing pyroptotic cell death. Hence, their involvement in cancer cell pyroptosis should be the focus of research attention. An in-depth investigation on the crosstalk of different cell death pathways will provide a new way to develop potentially effective cancer treatment.

##### 4.1.2.2 Negative regulation of GSDM family proteins

Epidermal growth factor receptor (EGFR) functions as a tumor promoter and is thus considered as a prospective therapeutic target for cancer treatment (129). Reportedly, cetuximab, a chimeric EGFR-targeted monoclonal antibody, inhibited the proliferation of EGFR-overexpressing triple-negative breast cancer (TNBC) cells (106). Accordingly, cetuximab might be a promising therapeutic agent for the treatment of EGFR-overexpressing TNBC. Moreover, it is proposed that the combination treatment of cetuximab and other agents could overcome acquired resistance to cetuximab in TNBC patients. miR-155-5p was found to be upregulated in TNBC cells (106). The miR-155-5p antagomir enhanced the cytotoxic effects of cetuximab on EGFR-overexpressing TNBC cells. Importantly, GSDME was the downstream target of miR-155-5p. As expected, cetuximab combined with the miR-155-5p antagomir fostered pyroptotic cell death in EGFR-overexpressing TNBC cells by enhancing the expression of GSDME-N and caspase-1. *In vivo* experimental results manifested that knockdown of miR-155-5p strengthened the anticancer activity of cetuximab in an EGFR-overexpressing TNBC xenograft mouse model by prompting cancer cell pyroptosis. Collectively, the combined use of cetuximab and the miR-155-5p inhibitor may be effective in TNBC therapy.

GSDM family proteins are the pore-forming executioners of pyroptosis. Activation of GSDM-mediated cancer cell pyroptosis could lead to tumor growth inhibition and enhanced anticancer immunity (130). GSDM-directed therapies may offer promising approaches for cancer immunotherapy. miRNAs are capable of reducing GSDM expression. Thus, the inhibitors of GSDM-targeted miRNAs may be useful for the treatment of cancer. Currently, there is limited information available concerning the regulation of GSDM expression by miRNAs. The identification and characterization of miRNAs that target GSDM proteins or their upstream/downstream effectors should be a focus of research in the future. Additional studies on miRNA-associated mechanisms regulating GSDM expression in both normal and cancerous cells will pave the way for screening and developing novel anticancer agents.

##### 4.1.2.3 Coordination of tumor suppressors

Tanshinone IIA promoted the pyroptosis of nasopharyngeal carcinoma (NPC) cells (107). Tanshinone IIA reduced the level of miR-125b and upregulated the expression of its target forkhead box P3 (FOXP3) in NPC cells. miR-125b overexpression or FOXP3 knockdown abrogated the pro-pyroptotic effect of tanshinone IIA on NPC cells, as evidenced by decreased levels of cleaved products of pyroptosis-related proteins including caspase-1, GSDMD-N, IL-1β and IL-18, as well as reduced release of ROS and LDH. Therefore, the miR-125b/FOXP3 signaling acted as a pyroptotic inhibitor in NPC. The contributory role of this signaling pathway in NPC development needs to be explored *in vivo*. FOXP3 is a member of the forkhead transcription factor family and plays a suppressive role in the immune system (131). The role of FOXP3 in carcinogenesis is conflicting. In some types of cancers, FOXP3 serves as a tumor promoter. While in some other types of cancers, it acts oppositely. The reason for the inconsistent results is yet to be clarified. FOXP3 was found to impede DNA damage repair in cancer cells (132), which might contribute to its participation in tanshinone IIA-induced pyroptosis. However, it is essential to characterize the exact mechanism underlying the role of FOXP3 in pyroptosis regulation.

Tumor suppressors have been found to stimulate pyroptotic cell death in cancer (88, 133). The mechanisms responsible for tumor suppressor-induced pyroptosis include activation of inflammasomes and domination of pyroptosis-relevant signaling pathways. Oncogenic miRNAs restrain cancer cell pyroptosis and facilitate cancer progression by downregulating tumor suppressors. The crucial role and underlying mechanism of the oncogenic miRNA/tumor suppressor axes in pyroptosis require comprehensive exploration. Increasing knowledge about miRNA-mediated regulation of pyroptosis will provide helpful information for further research of the mechanism and application of oncogenic miRNA-targeted therapies.

### 4.2 Regulation of pyroptosis by lncRNAs in cancer

#### 4.2.1 Fine-tuning of miRNA function

A growing body of evidence has indicated that lncRNAs act as competing endogenous RNAs (ceRNAs) to suppress miRNA function, thereby governing the expression of miRNA target genes. LncRNAs can modulate key components (e.g., inflammasomes and GSDM proteins) of the pyroptosis pathway by sponging miRNAs (**Table 1**). Moreover, the lncRNA/miRNA/mRNA ceRNA network is involved in regulating specific signal transduction cascades that are associated with pyroptosis. Therefore, lncRNAs are recognized as important players in cancer cell pyroptosis.

LINC00958 presented higher expression in oral squamous cell carcinoma (OSCC) cells than normal oral epithelial cells (108). LINC00958 overexpression remarkably enhanced OSCC cell proliferation by downregulating SIRT1 to decrease p53 expression. Ectopic expression of SIRT1 reversed the effects of LINC00958 on OSCC cell survival by upregulating p53. Intriguingly, LINC00958 reduced the expression level of miR-4306 that directly bound to AIM2. As a result, LINC00958 elevated the levels of caspase-1, IL-1β and IL-18, evidencing that LINC00958 induced AIM2-dependent pyroptosis. LINC00958 simultaneously exerted tumor-promoting and suppressive roles in OSCC. The pro-tumorigenic effect of LINC00958 seemed to take the leading position. These findings provided new insights into the exact mechanism related to OSCC progression and laid a theoretical foundation for future studies. The expression level of lncRNA small nucleolar RNA host gene 7 (SNHG7) was higher in hepatocellular carcinoma (HCC) tissues and liver cancer cells than adjacent normal tissues and normal liver epithelial cells (109). Overexpression of SNHG7 impeded NLRP3-dependent pyroptosis in liver cancer cells. SNHG7 functioned as a ceRNA of miR-34a that negatively modulated the expression of SIRT1. Silencing of SNHG7 lowered SIRT1 expression, but elevated the expression of NLRP3, caspase-1 and IL-β, eventually contributing to pyroptosis in liver cancer cells. miR-34a overexpression further strengthened SNHG7 knockdown-mediated pyroptosis. The SNHG7/miR-34a/SIRT1 axis played an important role in liver cancer progression *via* modulation of NLRP3-dependent pyroptosis. LINC00958 and SNHG7 had opposing roles in regulation of SIRT1 expression. LINC00958 promoted OSCC cell proliferation by downregulating SIRT1, while SNHG7 facilitated HCC cell survival by upregulating SIRT1. SIRT1 is a well-characterized member of the sirtuin family and plays a pivotal role in carcinogenesis (134). SIRT1 can function as a tumor promoter or suppressor, depending on their expression levels in cancer cells, actions on cell proliferation and death, as well as their effects on oncogenic and tumor-suppressive proteins (135). Future investigations are warranted to illustrate under which conditions SIRT1-regulating lncRNAs perform an enhancive or suppressive action on cancer cell pyroptosis.

LncRNA ADAMTS9-AS2 was downregulated in GC tissues and cells compared to their normal counterparts (110). LncRNA ADAMTS9-AS2 overexpression augmented the cytotoxic effects of cisplatin on cisplatin-resistant GC (CR-GC) cells. Moreover, upregulation of lncRNA ADAMTS9-AS2 increased NLRP3 expression and induced pyroptotic cell death in cisplatin treated CR-GC cells by targeting miR-223-3p. The pyroptosis inhibitor NSA could reverse the antagonistic effect of overexpressed lncRNA ADAMTS9-AS2 on CR-GC progression. Thus, lncRNA ADAMTS9-AS2 chemosensitized GC cells to cisplatin treatment by triggering miR-233-3p/NLRP3-mediated pyroptotic cell death.

Ionizing radiation (IR) could induce GSDME-executed pyroptosis in human CRC cells (21). LncRNA nuclear paraspeckle assembly transcript 1 (NEAT1) was highly expressed in response to IR and had the ability to increase GSDME expression by downregulating miR-448. Depletion of NEAT1 or ectopic expression of miR-448 suppressed the expression and activation of GSDME, leading to the inhibition of IR-induced pyroptosis. Accordingly, downregulation of NEAT1 rescued IR-caused reduction of cell viability in CRC cells. These findings demonstrated that lncRNA NEAT1 regulated IR-triggered pyroptosis in CRC cells by affecting the miR-448/GSDME axis. Altogether, the participation of NEAT1 in CRC pathogenesis involved a pyroptosis-associated mechanism.

LncRNA X inactivate-specific transcript (XIST) was overtly overexpressed in NSCLC tissues and cells, instead of their normal counterparts (111). Depletion of XIST enhanced NLRP3 inflammasome activation and ROS generation, contributing to pyroptotic cell death in NSCLC cells. This anticancerous effect could be abolished by the pyroptosis inhibitor NSA or the ROS scavenger N-acetyl cysteine (NAC). In terms of mechanism, XIST increased the expression of superoxide dismutase 2 (SOD2) by acting as a molecular sponge for miR-335. Oppositely, downregulated XIST promoted ROS-induced pyroptosis by lowering SOD2 expression in NSCLC cells. The miR-335 inhibitor counteracted the effects of XIST knockdown on ROS levels and cell pyroptosis, which could be reversed by synergistically depleting SOD2. Collectively, inhibition of XIST blocked NSCLC progression by activating the miR-335/SOD2/ROS signaling cascade to initiate pyroptotic cell death. XIST might be a potential therapeutic target for the treatment of NSCLC.

The expression of lncRNA HOXA transcript at the distal tip (HOTTIP) was markedly upregulated in ovarian cancer (OC) tissues and cells (112). HOTTIP silencing caused the repression of OC cell proliferation and the induction of NLRP1 inflammasome-dependent pyroptosis. Mechanistically, HOTTIP decreased the expression of miR-148a-3p and thus upregulated its downstream target protein kinase B2 (Akt2). miR-148a-3p stimulated the apoptosis signal-regulating kinase 1 (ASK1)/c-Jun N-terminal kinase (JNK) signaling by downregulating Akt2. Consistently, depletion of HOTTIP led to the activation of the ASK1/JNK signal transduction cascade, which could be reversed by Akt2 upregulation. Akt2 served as a signaling hub to prevent the phosphorylation of ASK1 and JNK, thereby downregulating the precursors of pyroptosis-related cytokines and inhibiting pro-inflammatory responses (136). The formation of NLRP1 inflammasome was associated with the ASK1/JNK signaling pathway (137). Accordingly, Akt2 overexpression abrogated the inhibitory effects of HOTTIP silencing on OC cell proliferation by blocking NLRP1 inflammasome formation, caspase-1 activation and the secretion of pro-inflammatory cytokines (112). To summarize, HOTTIP affected OC cell proliferation and pyroptosis *via* the miR-148a-3p/Akt2 axis.

LncRNAs play multifaceted roles in cancer cell pyroptosis by sequestering miRNAs. Targeting the lncRNA-miRNA interactions may contribute to improved anticancer therapies with decreased chance of off-target effects. However, the role of lncRNA/miRNA/mRNA ceRNA regulatory networks in cancer needs further study. Currently, our knowledge about lncRNAs remains insufficient due to their diversity and complexity. Substantial efforts are required to discover and characterize as-yet-unannotated functional lncRNAs in cancer. Notably, a single lncRNA could regulate the expression and activity of diverse miRNAs, thus influencing various key steps in the pyroptosis pathway, such as inflammasome formation, caspase-1 activation, GSDM cleavage and pro-inflammatory cytokine production. The multifarious functions of lncRNAs in regulation of pyroptosis are worthy of thorough investigation.

#### 4.2.2 Domination of inflammasome formation

Cisplatin treatment triggered NLRP3 inflammasome-dependent pyroptosis in TNBC cells (113). Remarkably, lncRNA maternally expressed gene 3 (MEG3) was upregulated in cisplatin-treated TNBC cells. Depletion of MEG3 was capable of abrogating cisplatin-induced NLRP3 inflammasome activation as reflected by downregulation of NLRP3, caspase-1 and GSDMD-N, as well as reduced secretion of IL-1β and IL-18. As expected, MEG3 knockdown promoted the proliferation and migration of cisplatin-treated TNBC cells by counteracting the pro-pyroptotic effect of cisplatin. These results revealed that MEG3 mediated cisplatin-induced pyroptosis in TNBC, implicating it as a potential target for developing new therapeutic avenues to treat TNBC. It remains unclear how MEG3 regulates the activation of NLRP3 inflammasome. Given that lncRNAs act as pivotal regulators of gene expression through diverse molecular mechanisms (138), the mode of action of MEG3 in cisplatin-induced pyroptosis is worthy of further investigation.

The oncogenic potential of XIST in NSCLC progression was previously investigated (114). XIST was dramatically upregulated in cisplatin-treated NSCLC tissues. XIST interacted with the TGF-β effector, mothers against decapentaplegic homolog 2 (SMAD2), and impeded its nuclear transfer. These events suppressed the transcription of both p53 and NLRP3. As expected, XIST knockdown could aggrandize cisplatin-induced pyroptosis in NSCLC cells by increasing the expression levels of NLRP3, caspase-1, IL-1β, IL-18 and GSDMD-N. SMAD2 knockdown could negate the inhibitory effects of XIST depletion on NSCLC cell proliferation. Accordingly, XIST deficiency suppressed the proliferation and enhanced cisplatin sensitivity of NSCLC cells partially by inducing pyroptotic cell death. The *in vivo* studies also showed that silencing of XIST potentiated cisplatin-induced pyroptosis in NSCLC cells, which led to the prevention of NSCLC xenograft growth. Regulation of the XIST/SMAD2/NLRP3 signaling cascade might provide novel therapeutic interventions to circumvent chemoresistance in NSCLC and perhaps other cancer types.

The expression of lncRNA growth arrest-specific transcript 5 (GAS5) was significantly reduced in OC tissues compared to matched adjacent normal tissues (115). LncRNA GAS5 knockdown markedly inhibited the expression of ASC, caspase-1, IL-1β and IL-18, and reduced LDH activity. Ectopic expression of lncRNA GAS5 exerted the adverse effect. Accordingly, lncRNA GAS5 inhibited OC cell proliferation by activating cell pyroptosis. The inflammasome inhibitor Vad-Fmk reversed GAS5-mediated tumor inhibition in OC xenograft nude mice. GAS5 could be utilized as a prospective therapeutic target for OC treatment. According to existing evidence, lncRNA GAS5 was proposed to control inflammasome formation and inflammatory responses by acting as a decoy for the glucocorticoid receptor (GR) (139, 140). Nevertheless, additional research efforts are essential to determine the precise role and mechanism of GAS5 in affecting inflammasome activation.

#### 4.2.3 Modulation of oncogenic proteins

The expression level of the tumor protein 63 (p63)-associated lncRNA RP1-85F18.6 was increased in CRC tissues and cells (116). Depletion of lncRNA RP1-85F18.6 could retard the proliferation and invasion of CRC cells. Importantly, knockdown of lncRNA RP1-85F18.6 enhanced LDH release, promoted GSDMD cleavage and cell membrane rupture, leading to CRC cell pyroptosis. Overexpression of lncRNA RP1-85F18.6 exerted the opposite roles. LncRNA RP1-85F18.6 was positively associated with the expression of the N-terminal truncated isoform of p63 (ΔNp63). ΔNp63 functions as an oncogene and can promote cancer cell proliferation by modulating specific transcriptional programs (141). Knockdown of ΔNp63 counteracted the pro-tumorigenic effects of overexpressed lncRNA RP1-85F18.6 (116). Thus, lncRNA RP1-85F18.6 inhibited pyroptosis, and promoted proliferation and invasion of CRC cells by modulating ΔNp63. It is intriguing whether lncRNA RP1-85F18.6 regulates ΔNp63 by interacting with particular proteins to enhance ΔNp63 stability. The detailed mechanism by which lncRNA RP1-85F18.6 alters ΔNp63 expression needs to be elucidated.

#### 4.2.4 Clinical significance of pyroptosis-related lncRNAs in cancer

**Figure 3:**
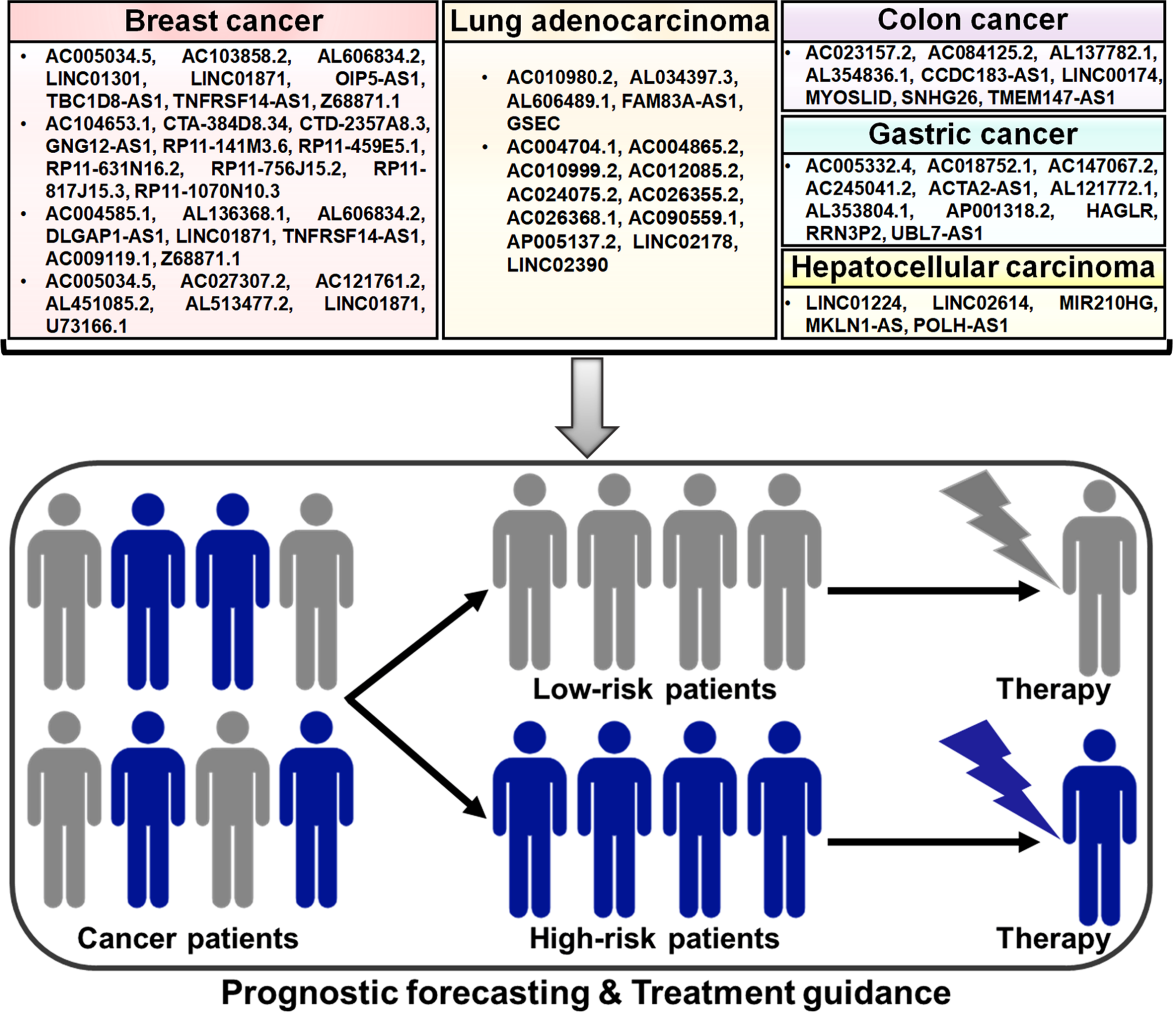
Potential clinical application of pyroptosis-related lncRNAs in cancer. Some prognostic models based on pyroptosis-related lncRNAs have been established, which can be useful in predicting the risk of poor clinical outcomes in patients with different cancers, including breast cancer, lung adenocarcinoma and gastrointestinal tumors. These pyroptosis-related lncRNA prognostic models may be a promising tool for assessing therapeutic response in cancer patients and thus offer helpful guidance for personalized treatment of cancers.

Compelling evidence has proven the important roles of lncRNAs in the process of pyroptotic cell death. LncRNAs affect various key hallmarks of cancer progression by acting directly or indirectly on the pyroptosis pathway. Pyroptosis-related lncRNAs may have potential utility as prognostic markers for risk stratification and treatment guidance in cancer patients (**Figure 3**). Pyroptosis-related lncRNAs have generated increasing interest in recent years due to their potential applications in the clinical practice.

##### 4.2.4.1 Breast cancer

Based on the transcriptomic and clinical data of BC patients from The Cancer Genome Atlas (TCGA) database, nine pyroptosis-related lncRNAs (AC005034.5, AC103858.2, AL606834.2, LINC01301, LINC01871, OIP5-AS1, TBC1D8-AS1, TNFRSF14-AS1 and Z68871.1) were identified for the prognostic signature, which divided BC patients into low- and high-risk groups (142). Univariate and multivariate Cox regression analyses showed that this lncRNA signature had potential prognostic value in predicting clinical outcome in BC patients. Further study revealed that low-risk BC might be an immunologically “hot” tumor. The pyroptosis-associated risk score had an inverse relationship with the levels of tumor-infiltrating lymphocytes (TILs, NK cells and T cells) and immune checkpoint molecules (e.g., BTLA, CTLA4, LAG3 and PD-L1). However, the infiltration levels of M2 macrophages and mast cells were remarkably higher in the high-risk group. It was likely that induction of pyroptosis might represent a promising therapeutic strategy for the improvement of immunotherapy efficacy in BC patients.

Ten pyroptosis-related lncRNAs (AC104653.1, CTA-384D8.34, CTD-2357A8.3, GNG12-AS1, RP11-141M3.6, RP11-459E5.1, RP11-631N16.2, RP11-756J15.2, RP11-817J15.3 and RP11-1070N10.3) were identified as independent predictors for overall survival in BC patients (143). BC patients were clustered into low- and high-risk groups based on the pyroptosis-related lncRNA risk model. The nomogram based on the risk model and clinicopathologic features displayed good performance in predicting prognosis in BC patients. Low-risk patients had more infiltration of antitumor immune cells, such as activated memory CD4+ T cells and CD8+ T cells, while high-risk patients had more B cells, CD8+ T cells, cytotoxic lymphocytes, M2 macrophages, NK cells and regulatory T (Treg) cells. The expression levels of T cell phenotypic and functional markers (e.g., CD4, CD8B and GZMB), activating immune receptors (CD27, CD40 and CD80) and immune checkpoint markers (CTLA4, PDCD1 and PD-L1) were elevated in the low-risk group compared with the high-risk group. In addition, the expression levels of RP11-1070N10.3, PR11-141M3.6 and RP11-817J15.3 were decreased, while RP11-459E5.1 was overtly upregulated in BC tissues compared to paired normal tissues. The expression level of RP11-1070N10.3 and RP11-817J15.3 was positively correlated with overall survival in BC patients, while that of RP11-459E5.1 was inversely associated with overall survival. This signature could be useful in screening BC patients who may benefit from immunotherapy.

Lv et al. (144) screened eight pyroptosis-related lncRNAs to construct a prognostic model. Among these lncRNAs, AC004585.1, AL136368.1, AL606834.2, DLGAP1-AS1, LINC01871 and TNFRSF14-AS1 were favorable prognosis factors, while AC009119.1 and Z68871.1 acted as predictive indicators for poor prognosis. BC patients were clustered into low- and high-risk groups. The risk score was proven to be an independent prognostic factor characterized by good predictive sensitivity and specificity. The infiltration levels of CD8+ T cells, neutrophils, NK cells, T helper (Th) cells and Treg cells were lower in the high-risk group than those in the low-risk group. Experimental results indicated that AC009119.1 and Z68871.1 were upregulated and other six lncRNAs were downregulated in the high-risk group. AC004585.1, AL606834.2 and LINC01871 were positively associated with the infiltration level of memory B cells. These results suggested that the risk model based on the eight pyroptosis-related lncRNAs would offer new perspectives for BC prognosis prediction.

Seven lncRNAs AC005034.5, AC027307.2, AC121761.2, AL451085.2, AL513477.2, LINC01871 and U73166.1 were screened to develop a prognostic prediction model due to their significant correlation with overall survival of BC patients (145). This model was an independent prognostic factor in BC patients characterized by high stability and efficacy. High expression level of AC121761.2, AL451085.2, AL513477.2, LINC01871 and U73166.1 was associated with better overall survival, but that of AC005034.5 and AC027307.2 was linked to worse overall survival. Immune infiltration analysis indicated that high-risk patients were prone to have high infiltration levels of γδ T cells and Th2 cells and low levels of CD8+ T cells, cytotoxic cells and Th cells. The expression level of AC027307.2 was verified to be elevated while that of AC121761.2 was reduced in BC cells relative to normal breast epithelial cells. The identified pyroptosis-related lncRNAs might be potential biomarkers and therapeutic targets in BC. However, the roles of these lncRNAs in BC progression need to be exhaustively investigated in future studies.

##### 4.2.4.2 Respiratory system tumor

A pyroptosis-related lncRNA signature for predicting prognosis in lung adenocarcinoma (LUAD) was set up (117). Five lncRNAs AC010980.2, AL034397.3, AL606489.1, FAM83A-AS1 and GSEC were incorporated into this signature, which separated LUAD patients into low- and high-risk groups. The predictive accuracy of this prognosis signature was determined by the receiver operative characteristic (ROC) analysis. High-risk scores tended to be correlated with decreased levels of immune cell infiltration and increased tumor mutation burden. Moreover, GSEC exhibited upregulation in LUAD tissues and cells relative to non-cancerous tissues and normal lung epithelial cells. Upregulated GSEC acted as a predictive indicator of poor overall survival in LUAD patients. Further experimental research showed that GSEC knockdown enhanced the expression of NLRP3 and caspase-1, which might contribute to pyroptosis induction and growth inhibition in LUAD cells. Thus, the five pyroptosis-related lncRNA signature demonstrated good performance in prognostic forecasting and might serve as a clinically available guide for personalized treatment of LUAD patients. Eleven pyroptosis-related lncRNAs AC004704.1, AC004865.2, AC010999.2, AC012085.2, AC024075.2, AC026355.2, AC026368.1, AC090559.1, AP005137.2, LINC02178 and LINC02390 were incorporated into a prognostic signature (146). The lncRNA signature was validated to have significant prognostic value in LUAD. The signature-identified risk score was inversely associated with the expression levels of immune checkpoints (e.g., CD47, PD-1 and PD-L1), implying that the lncRNA prognostic model might provide guidance for future immunotherapy in LUAD patients. Importantly, the risk score might act as an independent factor for predicting prognosis in LUAD. The expression levels of AC004704.1 and AC024075.2 were decreased, while the other nine lncRNAs were upregulated in LUAD cells compared with normal lung epithelial cells. Nevertheless, the mechanisms of these pyroptosis-related lncRNAs in the occurrence and development of LUAD deserve additional study. The clinical value of this prognostic signature awaits further corroboration in larger cohorts of LUAD patients.

##### 4.2.4.3 Gastrointestinal tumors

Liu et al. (147) identified 20 pyroptosis-related lncRNAs that showed differential expression patterns between colon cancer tissues and normal colon tissues. A nine lncRNA signature comprising AC023157.2, AC084125.2, AL137782.1, AL354836.1, CCDC183-AS1, LINC00174, MYOSLID, SNHG26 and TMEM147-AS1 was set up, and it classified all patients into low- and high-risk groups. The signature-identified risk score was a useful indicator for predicting the prognosis of patients with colon cancer. High-risk patients had higher expression level of immune checkpoints (CTLA4, PD-1 and PD-L1) than the low-risk group. The nine lncRNA signature might have valuable clinical application for accurate prognostic prediction, which could assist the development of specialized treatment regimens for patients with colon cancer.

Pyroptosis-related lncRNAs AC005332.4, AC018752.1, AC147067.2, AC245041.2, ACTA2-AS1, AL121772.1, AL353804.1, AP001318.2, HAGLR, RRN3P2 and UBL7-AS1 were selected for establishment of a prognostic model for separating low- and high-risk GC patients (148). This risk model showed favorable performance in assessing the survival outcomes of GC patients. The risk score was superior in prognostic prediction compared with clinical characteristics, such as age and tumor stage. The pyroptosis-related lncRNA signature might offer valuable clinical application for better prognostic forecasting in GC. Low-risk patients had more infiltration of NK cells and T cells, whereas high-risk patients showed more abundant infiltration of macrophages, activated mast cells, monocytes and neutrophils. High-risk patients displayed high sensitivity to chemotherapies, such as dasatinib and imatinib. In addition, the expression levels of immune checkpoints (e.g., CD160, CTLA4, TNFRSF14 and TNFSF15) were increased in the low-risk group compared to the high-risk group. Collectively, the risk model might be useful in evaluating the immune status of GC patients and provide guidance for treatment assignments.

Five pyroptosis-related lncRNAs (LINC01224, LINC02614, MIR210HG, MKLN1-AS and POLH-AS1) were upregulated in HCC tissues in comparison with paired adjacent normal tissues (149). The pyroptosis-related lncRNA risk model showed favorable performance in predicting prognosis of HCC patients. The nomogram based on the risk model and clinicopathologic features might be more efficient in predicting the clinical outcome of HCC patients than the use of individual clinical characteristics. The risk score was negatively associated with the infiltrating levels of B cells, NK cells and plasmacytoid DCs. High-risk patients had increased expression levels of immune checkpoints (e.g., CD28, PD-L1 and TNFRSF14) and showed enhanced responses to chemotherapy. The risk model could be employed to drive treatment decision making in an individualized manner to direct improved therapies in HCC patients. Further investigation is required to delve into the complex interplay between pyroptosis-related lncRNAs and tumor immunomodulation during HCC development.

Pyroptosis-related lncRNA prognostic models could open up new perspectives for advancing personalized treatment for cancer patients. However, there is limited research on their potential clinical implication. More and more lncRNAs interfering with the pyroptosis pathway have been discovered in cancer. It is a necessity to identify and develop novel cancer biomarkers based on pyroptosis-related lncRNA expression profiles. So far, the original data for constructing pyroptosis-related lncRNA signatures is mainly retrieved from the TCGA database. The lncRNA risk models need to be substantiated for precision and reliability in other external datasets. The genuine role and underlying mechanisms of pyroptosis-related lncRNAs in cancer await in-depth investigations. Importantly, large-scale multicenter prospective studies should be undertaken to explore the prognostic value of pyroptosis-related lncRNA signatures.

### 4.3 Regulation of cancer cell pyroptosis by circRNAs

CircRNAs are emerging as crucial regulators of the pyroptosis pathway in cancer (**Table 1**). CircNEIL3 was remarkably downregulated in irradiated LUAD cells compared with non-irradiated LUAD cells (23). CircNEIL3 deficiency fostered irradiation-induced pyroptotic cell death in LUAD. By contrast, circNEIL3 upregulation exerted the opposite effects by inhibiting the activity of caspase-1 to cleave GSDMD into pore-forming GSDMD-N fragment. CircNEIL3 acted as a miR-1184 sponge to coordinate the expression of the DNA helicase petite integration factor 1 (PIF1). miR-1184 overexpression not only promoted the release of LDH, IL-1β and IL-18, but also enhanced the expression of active caspase-1 and GSDMD-N in LUAD cells, which were dramatically abrogated by ectopic circNEIL3 expression. Moreover, PIF1 ablation abolished the anti-pyroptotic effects of circNEIL3 on LUAD cells. PIF1 helicase has been associated with DNA replication and damage. The circNEIL3/miR-1184/PIF1 axis could regulate DNA damage repair by downregulating the γ phosphorylated form of the histone H2AX (γH2AX), a key marker of DNA damage. It has been reported that DNA damage activates AIM2 inflammasome, thereby triggering GSDMD-executed pyroptosis and pro-inflammatory cytokine production (150). Consistently, PIF1 knockdown promoted the formation of AIM2 inflammasome in irradiated LUAD cells. The circNEIL3/miR-1184/PIF1 axis inhibited LUAD cell pyroptosis by preventing DNA damage-induced AIM2 inflammasome activation. CircNEIL3 knockdown was effective in enhancing the susceptibility of LUAD cells to radiation therapy and suppressing tumor growth in a nude murine LUAD xenograft tumor model. Collectively, circNEIL3 knockdown-mediated pyroptosis might provide a novel therapeutic strategy for improving the efficiency of radiotherapy in LUAD.

Cobalt chloride (CoCl_2_) treatment induced the upregulation of hypoxia-inducible factor-1α (HIF-1α), which mimicked the intracellular bioenergetic variations of tumor hypoxia (151). CircPUM1, a circRNA derived from the *PUM1* gene, was overtly upregulated following HIF-1α accumulation in CoCl_2_-treated ESCC cells (118). CircPUM1 promoted the growth of ESCC cells *in vitro* and *in vivo*. CircPUM1 knockdown elevated the expression of caspase-3 and GSDME-N in ESCC cells. Consequently, circPUM1 silencing promoted LDH release and induced ESCC cell pyroptosis. The AMP-activated protein kinase (AMPK) pathway was activated in response to energy stress and could trigger caspase-3/GSDME-mediated pyroptosis in cancer (152, 153). As expected, circPUM1 deficiency increased the phosphorylation of AMPK in ESCC cells. The AMPK inhibitor dorsomorphin dihydrochloride inhibited GSDME cleavage in circPUM1-knockdown ESCC cells, while elevated AMPK activity facilitated GSDME activation. CircPUM1 was capable of suppressing caspase-3/GSDME-mediated ESCC cell pyroptosis by targeting the AMPK signaling pathway. Intriguingly, circPUM1 enhanced oxidative phosphorylation for ATP production and reduced ROS production by acting as a scaffold for the interaction between ubiquinol-cytochrome c reductase core proteins (UQCRC1 and UQCRC2) in mitochondrial complex III. Increased levels of intracellular ROS and caspase-3 were able to initiate GSDME-mediated pyroptosis in cancer (154). It was reasonable to infer that circPUM1 exerted an inhibitory effect on ESCC cell pyroptosis through regulation of intracellular ATP level and ROS generation. The complicated mechanisms underlying the regulatory role of mitochondria-located circPUM1 in pyroptosis deserve further investigation. CircRNAs have pivotal functions as modulators of pyroptosis, yet there are limited studies investigating the relationship between circRNAs and pyroptosis. Thus, intensive research efforts are needed to characterize the perplexing roles of circRNAs in pyroptosis regulation.

## 5 Conclusions and future perspectives

As an immunogenic cell death pathway, pyroptosis plays a key role in tumor suppression. Pharmacological induction of pyroptotic cell death will offer an alternative therapeutic strategy for the clinical treatment of various cancers. At present, there are many gaps in fundamental knowledge regarding the genuine role of pyroptosis in cancer. What is more, it is of paramount importance to clarify the detailed regulatory mechanisms of pyroptosis during cancer development. Given that excessive pyroptosis may be detrimental, the sophisticated mechanisms responsible for the initiation and shutdown of the pyroptosis pathway must be elucidated through additional studies. Inflammatory factors released from pyroptotic cancer cells play a crucial role in antitumor immunity. The combination of pyroptosis-activating agents and immunotherapy may strengthen anticancer benefits. Paradoxically, the occurrence of pyroptosis gives rise to the discharge of tumor-promoting molecules, such as IL-1β and IL-18. Continuous studies will be necessary to investigate the fate of pyroptotic cell-derived inflammatory factors. Chemotherapeutic agents such as doxorubicin and paclitaxel are supposed to have enormous anticancer potentials due to their pyroptosis-inducing effects (155). However, cancer cells can always develop resistance to these chemotherapeutic agents (156, 157), which remains to be addressed in further studies. By and large, more in-depth investigations centering upon molecular mechanisms of pyroptosis are critical to open up new therapeutic opportunities for the intervention of multiple cancers.

Recently, ncRNAs have been acknowledged as master regulators of pyroptosis in cancer, adding a new layer of complexity to the regulatory mechanisms of pyroptosis. Various types of ncRNAs, such as miRNAs, lncRNAs and circRNAs, are involved in pyroptosis regulation. However, there are limited studies reporting the relationship between ncRNAs and pyroptosis, and many hurdles remain to be overcome. First, the majority of studies that address the role of ncRNAs in pyroptosis regulation were done *in vitro* or in mice. Mice models cannot fully recapitulate the complexities of human cancers. Thus, further clinical studies are required before extending the conclusion from mice to humans. Secondly, owing to the great diversity of ncRNAs, it will be challenging to disclose their exact mechanisms of action in modulating pyroptosis. Ongoing studies are required to confirm whether other types of ncRNAs have the ability to modulate the pyroptosis process in cancer, in addition to miRNAs, lncRNAs and circRNAs. In some cases, multiple abnormally expressed ncRNAs may exert synergetic or antagonistic effects on certain pyroptosis pathways. Therefore, it is arduous to ascertain which ncRNA occupies the leading position in pyroptosis regulation, making it infeasible to specifically induce pyroptosis in cancer cells through modulation of ncRNA activity. Thirdly, the implication of ncRNAs in cell death mechanisms deserves more explorative research. Since studies have indicated that ncRNAs serve as key players in the apoptotic and autophagic pathways, some pyroptosis-regulating ncRNAs may simultaneously fine-tune these cell death pathways. The crosstalk between cell death pathways regulated by ncRNAs deserves further investigation. Fourthly, the mechanisms of ncRNAs in regulating pyroptosis are largely equivocal. Aside from their influence on gene expression, ncRNAs can modulate chromatin dynamics and alternative splicing, interact with proteins and even produce functional peptides. It is intriguing whether the regulatory effects of ncRNAs on pyroptosis could be attributable to their actions mentioned above. Several types of ncRNAs, such as lncRNAs and circRNAs, may modulate each other by acting as ceRNAs to compete for interacting with shared miRNAs. The pyroptosis-associated ceRNA regulatory networks in cancer are far from fully clarified. The impact of ncRNAs on pyroptosis in cancer is complicated and warrants special attention. Finally, current investigation of pyroptosis-related ncRNAs is at the infancy stage, which hinders the translation of research findings. Pyroptosis-related ncRNAs have the potential to be used as therapeutic targets or molecular biomarkers in cancer. An in-depth understanding of pyroptosis-regulating ncRNAs is of great importance before preclinical findings can be translated into more effective anticancer treatments and more accurate prognostic systems for cancer patients. Targeting pyroptosis-regulating ncRNAs may present an adjuvant to traditional anticancer therapies. Regulation of ncRNA expression can be achieve *via* several methodologies, including ncRNA mimics, antisense oligonucleotides and specific chemicals. Future research efforts should focus on identifying chemicals with the function of regulating the expression of pyroptosis-related ncRNAs. Recent technological advancements have enabled the synthesis and manufacture of ncRNA-based molecules for utilization in preclinical studies. Since synthetic ncRNA mimics and inhibitors are easily degraded *in vivo* and may fail to reach target tissues, chemical modifications have been tailored to improve the stability of synthetic ncRNA mimics/oligonucleotides and to diminish potential off-target effects. Artificial delivery systems, such as exosomes, lipid nanoparticles and polymer-based formulations, have also been developed to convey fragile synthetic ncRNAs to the destination site. However, the impact of structural modifications on the biological activity of ncRNA-based molecules needs extensive study. The safety and efficiency of ncRNA delivery platforms should be adequately investigated. Furthermore, it is worthwhile noting that the molecular features and functions of pyroptosis-related ncRNAs may vary in different cancers. The effectiveness of ncRNA-based therapeutics in each cancer type must be clarified. Dysregulated pyroptosis-related ncRNAs have shown promise as prognostic markers in cancer. Isolation, processing and detection approaches of biological samples may have an impact on the performance accuracy of pyroptosis-related ncRNA-based prognostic models. Standardized procedures should be established to validate the prognostic value of pyroptosis-related ncRNAs. The use of pyroptosis-related ncRNA signatures concurrent with conventional cancer biomarkers (e.g., CA19-9 and CEA) could be a promising predictive tool that allows for better risk stratification and treatment choices for cancer patients. Above all, large-scale clinical studies are critically needed to evaluate the clinical relevance of pyroptosis-related ncRNAs. Despite the aforementioned problems to be solved, the auspicious and significant prospect of pyroptosis-regulating ncRNAs is worthy of the wait.

## Author contributions

MW and PL conceived this article. MW drew the figures and wrote the manuscript. YZ, WC and LZ collected the related papers and revised the manuscript. KS and PL helped to edit the manuscript. All authors have read and approved the final version of the manuscript.

## Funding

This work was supported by the Natural Science Foundation of Shandong Province, China (Grant No. ZR2021MH018).

## Conflict of interest

The authors declare that the research was conducted in the absence of any commercial or financial relationships that could be construed as a potential conflict of interest.

## Publisher’s note

All claims expressed in this article are solely those of the authors and do not necessarily represent those of their affiliated organizations, or those of the publisher, the editors and the reviewers. Any product that may be evaluated in this article, or claim that may be made by its manufacturer, is not guaranteed or endorsed by the publisher.
